# Human tick biting and tick-borne disease risk in Türkiye: Systematic review

**DOI:** 10.1371/journal.pntd.0013092

**Published:** 2025-06-09

**Authors:** Salar Zarrabi Ahrabi, Fatihan Pınarlık, Gürkan Akyıldız, Mert Kuşkucu, Sırrı Kar, Önder Ergönül, Ayşen Gargılı Keleş, on behalf of TEH Vector-Borne Infections Study Group

**Affiliations:** 1 Department of Basic Health Science, Health Sciences Faculty, Marmara University, Istanbul, Türkiye; 2 Koc University Isbank Center for Infectious Diseases (KUISCID), Istanbul, Türkiye; 3 Graduate School of Health Sciences, Koc University, Istanbul, Türkiye; 4 Department of Medical Microbiology, School of Medicine, Koc University, Istanbul, Türkiye; 5 Department of Biology, Faculty of Arts and Science, Namık Kemal University, Tekirdağ, Türkiye; 6 Department of Infectious Diseases and Clinical Microbiology, School of Medicine, Koc University, Istanbul, Türkiye; Egerton University, KENYA

## Abstract

Ticks serve as significant vectors for over 100 pathogens, many of which pose serious health risks to humans. Türkiye’s diverse tick species and ideal ecological conditions facilitate their proliferation. Following the emergence of the Crimean-Congo Hemorrhagic Fever (CCHF) epidemic in 2004, tick-borne diseases have become a critical public health concern. This systematic review was conducted to identify the dominant tick genera and species responsible for human bites and tick-borne diseases. PubMed, Cochrane, Scopus, Web of Science, Medline, Tübitak TR Dizin, Dergi Park databases were searched following PRISMA guidelines, and the last search was performed on 11 October 2024. Studies reporting human-biting ticks were included and studies that do not report tick species or number of ticks were excluded. A novel quality assessment scale was developed by Türkiye Infectious Diseases (TEH) Vector-Borne Infections Study Group and used for risk of bias assessment. The total number of ticks and percentages were calculated. A total of 24 studies documented 53,879 ticks, 96.60% of which were identified at the genus or species level. The most prevalent genera were *Hyalomma* (46.99%) and *Ixodes* (28.49%), followed by *Rhipicephalus* and *Haemaphysalis*. Notably, immature forms of *Hyalomma* spp., particularly nymphs, accounted for the highest proportion of bites (22.65%). The findings highlight *Hyalomma* and *Ixodes* as primary vectors for major diseases in Türkiye, with *H. marginatum* playing a central role in seasonal outbreaks of CCHF in rural Anatolia and *Ixodes* spp. linked to Lyme disease. Türkiye’s geographic and climatic diversity, along with factors such as migratory bird routes, facilitates the distribution of ticks and the emergence of novel tick-borne diseases. Despite the notable risks, inconsistent tick identification and reporting impede accurate assessment and management. Standardized methodologies and comprehensive reporting systems are strongly recommended to better address the public health risks posed by tick-borne diseases.

## Author summary

Ticks have become a critical public health concern after the emergence of Crimean Congo Hemorrhagic Fever, a deadly tick-borne disease, in Türkiye. This systematic review shows the distribution of human-biting ticks in Türkiye according to their genera and species. We report that *Hyalomma* and *Ixodes* as primary vectors for disease. These ticks are known disease spreaders for Crimean Congo Hemorrhagic Fever and Lyme disease. Depending on the ecological conditions in the following years, new vector-borne infections may occur. We recommend standardized methodologies and reporting systems to avoid false identification and reduce unidentified tick species in future reports because they affect the assessment and management of the patients having tick bites.

## Introduction

To date, 1150 valid species of ticks have been determined (949 *Ixodidae*, 200 *Argasidae* species, and *Nuttalliellidae* monotypic) [1]. However, only 10% of these species can act as vectors and transmit more than 100 pathogens including the Crimean Congo hemorrhagic fever (CCHF) virus [2]. While many ticks prefer specific hosts, they can feed on alternative hosts in unfavorable conditions [3]. Several tick species in Türkiye can transmit pathogens that cause human disease or have been identified as vectors. In previous studies, the genera *Hyalomma*, *Ixodes*, *Haemaphysalis*, *Dermacentor*, *Rhipicephalus, Argas*, and *Ornithodorus* have been reported in the region as vectors or potential vectors [4,5].

Tick-borne diseases have been a critical public health problem in Türkiye, particularly over the last two decades. After the first reports of CCHF cases in the early 2000s, tick-borne diseases have become a research priority [6].

Due to ecological and geographical dynamics, Türkiye has potential elements for vectors, particularly ticks, to spread diseases and increase biorisks [7]. There are numerous cases and reports of tick-borne diseases associated with tick bites, such as CCHF, Lyme, and rickettsiosis in Türkiye [8–10]. Türkiye has a high potential for ticks to spread, reproduce, increase in population, and pose risks, due to regional dynamics. For example, Türkiye’s geographic region has one of the main transit routes of migratory birds, which has the potential to carry ticks between various areas in the region. Moreover, diverse climatic types and vegetation facilitate populations of different tick species to establish in various regions [11]. When considering all these elements, Türkiye has become a high-risk country for tick-borne diseases.

Türkiye is home to a diverse range of tick species, which pose a significant public health risk due to the proximity of ticks to human populations. Individuals employed in agriculture, animal husbandry, slaughterhouses, and the healthcare sector are particularly vulnerable to tick bites and the diseases transmitted by these ectoparasites. The country’s rich vegetation, especially in the Marmara and Black Sea regions, creates ideal habitats for various tick species that can affect humans. These areas are also densely populated and popular for recreational activities, further increasing the risk of tick bites among the general population. As a result, a large portion of Türkiye’s population is exposed to the potential threat of tick-borne diseases [11,12]. To provide a better understanding of the tick-borne disease risk in Türkiye, we conducted this systematic review and identified the dominant tick genera and species responsible for human bites and tick-borne diseases.

## Methods

### Study design

Preferred Reporting Items for Systematic Reviews and Meta-Analyses (PRISMA) guidelines (S1 Appendix) were followed in the systematic review [13].

### Existing reviews

Previously published systematic reviews and meta-analyses on human-biting ticks in Türkiye were searched by two authors (SZA and FP) independently. The search through PubMed, Cochrane, Scopus, Web of Science, Medline, Tübitak TR Dizin, Dergi Park databases yielded no similar review.

### Search strategy

A comprehensive literature search through PubMed, Cochrane, Scopus, Web of Science, Medline, Tübitak TR Dizin, Dergi Park databases was conducted to collect studies reporting human tick biting, ticks, and tick-borne diseases in Türkiye. Tübitak TR Dizin and Dergi Park databases were intentionally included, as these databases contained the studies published in Turkish but not indexed in international databases. Only the reports that include data from Türkiye were selected without language restriction. The keywords in the search string were “tick-borne disease”, or “tick-borne diseases”, “tick-borne infection”, “tick bite”, or “tick bites”, “tick biting”, “tick infestation”, and “Türkiye”, or “Turkey”. The last database search was performed on October 11^th^, 2024.

### Selection criteria

Article titles were independently screened by two authors (SZA and FP) and selected studies were assessed for eligibility based on their abstracts. The studies that do not report tick bites and tick-borne diseases were excluded. Full-text screening was conducted for articles that were approved by at least one author. The inclusion criteria in full-text screening were reporting human-biting ticks and including data from Türkiye. The articles that did not give an exact number of ticks, did not report any data regarding Türkiye or did not report species-level tick identification were excluded. Although the identified ticks in some of the studies did not overlap with previous reports and they were inconsistent with the tick fauna of Türkiye, these studies were not excluded due to misdiagnosis of tick species. However, we recommend standardized methods for tick identification and reporting. The number of human-biting ticks and their identified genera and species were extracted by two authors (SZA and FP), and later cross-confirmed by a third author (AGK).

### Quality assessment

A quality assessment scale was developed by Türkiye Infectious Diseases (TEH) Vector-Borne Infections Study Group for reporting vectors. A consortium of veterinaries, parasitologists, virologists, infectious diseases doctors, and health sciences specialists determined the priority concerns for studies reporting vectors and vector-borne infections. TEH Quality Assessment Scale highlights the necessity of standardized methods for tick identification and reporting. It is a novel tool for bias assessment and it provides a standard methodology for future studies reporting vectors. It has six questions and examines a study reporting vectors in three domains: sample collection (Q1), identification of species (Q2-4), and reporting (Q5-6) (Table 1). Out of a total possible score of seven, studies scoring 1–3 are considered to have a high risk of bias, a score of 4–5 is an intermediate risk of bias, and a score of 6–7 is rated as a low risk of bias. Selected articles were independently evaluated by two authors (SZA and FP).

**Table 1:** TEH Quality Assessment Scale for reporting vectors.

Questions	Score
**Q1:** Has the study specified the years the samples were collected?	
Yes	1
No	0
**Q2:** Are the tick identification references mentioned in the material and methods?	
Yes	1
No	0
**Q3:** Have the reports identified the ticks to species or genus and unidentified ticks?	
The samples were identified to the species level	2
The samples were identified to the genus level	1
The samples were not identified	0
**Q4:** Are samples identified in terms of life stage?	
Yes	1
No	0
**Q5:** Does the study only focus on human tick-biting cases?	
Yes	1
No	0
**Q6:** Does the study report the exact number of collected samples?	
Yes	1
No	0
**Total Score**	…/7

### Data synthesis and analysis

The number of ticks and data regarding genus and species level identification reported in the selected studies were assessed in this review. To ensure consistency in the extraction of tick species data, two authors (SZA and FP) entered the number of ticks and cross-checked the data’s accuracy. The total number of ticks and percentages were calculated both at the genus and species level to examine the prevalence of different human-biting tick species. The spatial distribution of ticks was unfortunately not reported in some of the selected studies; therefore, this analysis could not be performed.

## Results

### Tick-biting cases on humans in Türkiye

**Fig 1:**
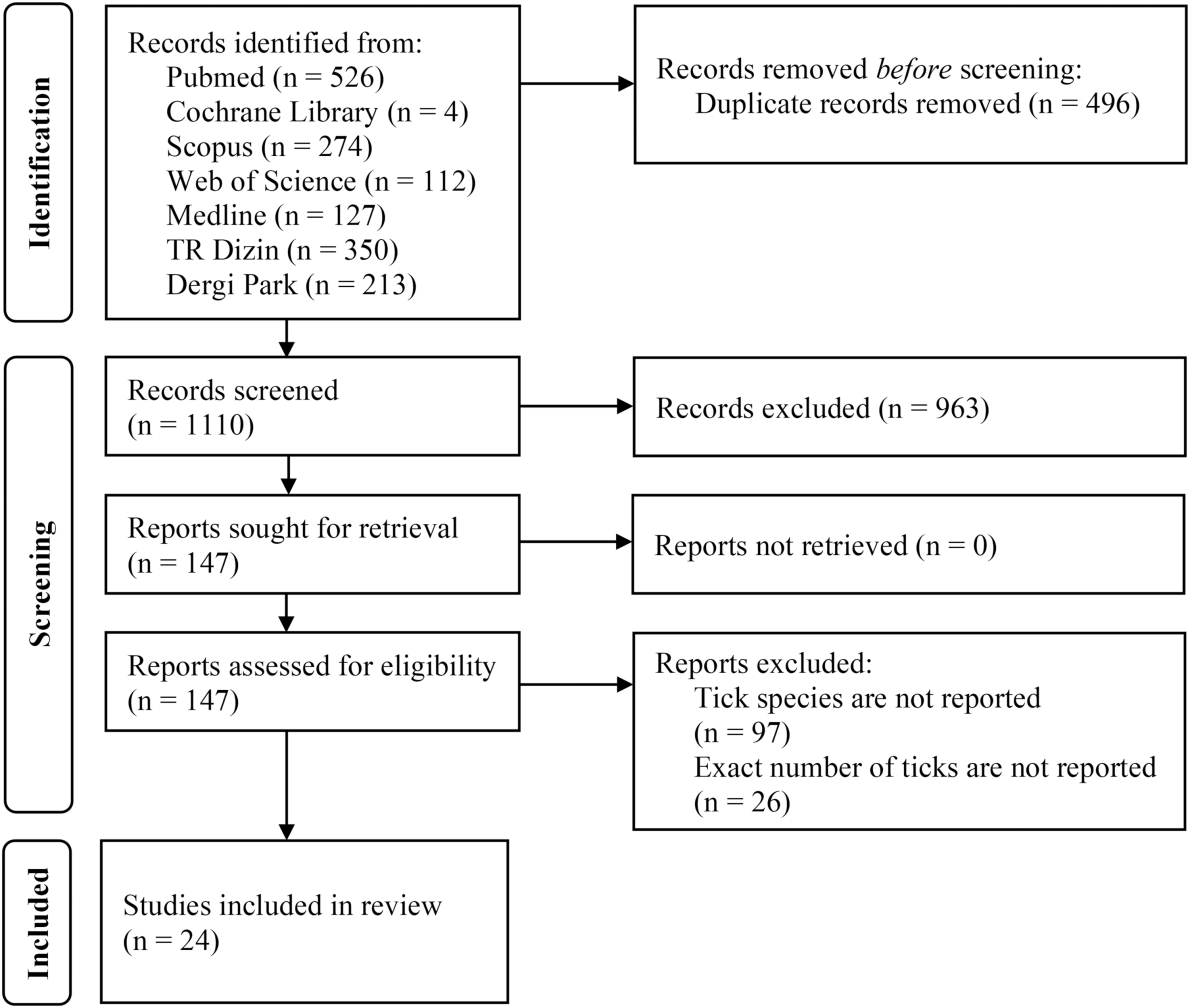
PRISMA flow diagram of the study selection process.

**Fig 2:**
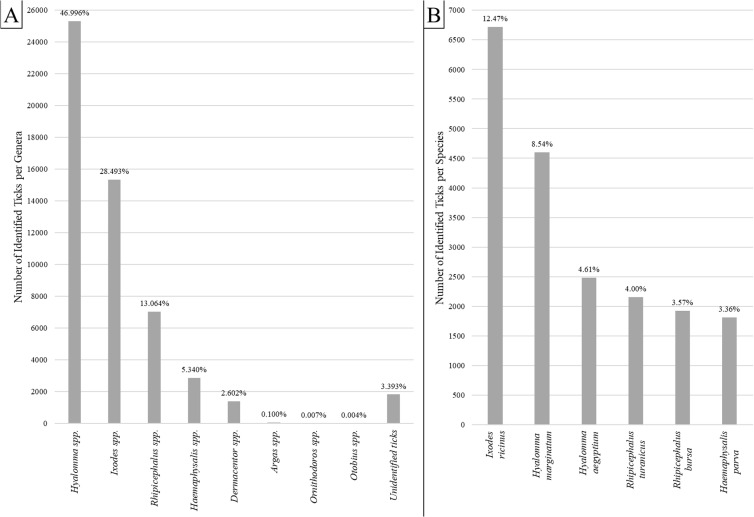
Distribution of identified tick genera **(A)** **and species** **(B)**. Percentages are labeled on top of columns.

After our literature search, 24 articles were included in the systematic review (Fig 1). Between 2008 and 2021, 53,879 ticks with reported genus or species were documented in the literature. In Türkiye, 52,051 (96.60%) ticks were identified at the level of genus or species and 24,602 (45.51%) were categorized at the species level (Table 2, S2 Appendix). In recent years, 12 *Hyalomma* species, seven *Ixodes* species, six *Haemaphysalis* species, five Rhipicephalus species, and four *Dermacentor* species were reported (S2 and S3 Appendicies). The most common species were *Hyalomma* spp. (46.99%) and *Ixodes* spp. (28.49%). Other genera include *Rhipicephalus* spp. (13.06%), *Haemaphysalis* spp. (5.34%), *Dermacentor* spp. (2.60%), *Argas* spp. (0.1%), *Ornithodoros* spp. (0.007%), and *Otobius* spp. (0.004%). Furthermore, 1828 ticks (3.39%) could not be identified in the selected studies and they are referred to as “unidentified ticks” (Fig 2A).

**Table 2:** Tick collection periods and number of identified ticks.

No	Tick Collection Period	Identified Ticks	References
1	2006	1054	[14]
2	2007	1478	[15]
3	2007	3121	[16]
4	2007	2664	[17]
5	2007	2610	[4]
6	2008	12	[18]
7	2008	179	[19]
8	2008	5999	[20]
9	2008	1925	[21]
10	2006-2008	391	[22]
11	2009	85	[23]
12	2009	104	[24]
13	2009	91	[25]
14	2009	2110	[26]
15	2008-2009	5094	[27]
16	2008-2009	241	[28]
17	2008-2009	1237	[29]
18	2007-2010	247	[30]
19	2006-2011	21198	[5]
20	2011	1468	[31]
21	2012-2013	169	[12]
22	2014	322	[32]
23	2015	152	[33]
24	2020-2021	100	[34]

Analysis of all tick bites reveals that more than 96% of human tick bites involve hard ticks. The immature form of *Hyalomma* spp. nymph is the most prevalent, accounting for 22.01% of tick bites. This is followed by the immature form of *Ixodes* spp. nymph, representing 14.04% of tick bites on humans.

A survey of the mature forms of species across several genus groups reveals that *Ixodes ricinus* is the most common species among mature ticks biting humans, accounting for 44% of the mature ticks in the *Ixodes* spp. genus. In the *Hyalomma* group, *Hyalomma marginatum* was the most frequently reported species, responsible for 18% of human tick bites. Within the *Rhipicephalus* spp. group, *Rhipicephalus turanicus* was the most reported species, comprising 31% of the cases. The highest percentage within a single group was observed in *Dermacentor marginatus*, which accounted for more than 71% of the mature ticks. In the *Haemaphysalis* spp. genus group, *Haemaphysalis parva* was the most reported species, responsible for about 63% of human bites. Among soft ticks, *Argas persicus* with 38% was the most frequently reported species of soft ticks to bite humans (Fig 2B).

### Risk of bias assessment

Among selected studies, 16 had a low risk of bias and 8 had an intermediate risk of bias (Table 3). All studies except two identified ticks on the species level, and 15 studies were only focused on human tick-biting cases while six failed to report the reference for identification (Table 3).

**Table 3:** TEH quality assessment score of selected articles.

Reference	Q1	Q2	Q3	Q4	Q5	Q6	Total Score
[14]	1	1	2	1	0	1	6
[15]	1	1	2	1	1	1	7
[16]	1	1	2	1	1	1	7
[17]	1	1	2	1	1	1	7
[4]	1	1	2	1	1	1	7
[18]	1	0	2	1	0	1	5
[19]	1	0	2	1	0	1	5
[20]	1	1	1	0	1	1	5
[21]	1	1	1	0	0	1	4
[22]	1	1	2	1	1	1	7
[23]	1	1	2	0	1	1	6
[24]	1	0	2	1	0	1	5
[25]	1	1	2	1	1	1	7
[26]	1	1	2	1	1	1	7
[27]	1	1	2	1	1	1	7
[28]	1	0	2	1	0	1	7
[29]	1	1	2	1	1	1	7
[30]	1	0	2	1	0	1	5
[5]	1	1	2	1	1	1	7
[31]	1	1	2	1	1	1	7
[12]	1	1	2	1	1	1	7
[32]	1	1	2	0	0	1	5
[33]	1	1	2	1	1	1	7
[34]	1	1	2	0	0	1	5

## Discussion

### Risk of ticks infesting humans in Türkiye

*Hyalomma* ticks, primarily three-host parasites, undergo three developmental stages — larval, nymphal, and adult — in the environment, each seeking specific hosts. The larvae and nymphs typically target small mammals, birds, and reptiles, while adult ticks are more likely to seek larger hosts such as cattle, sheep, and humans. Depending on ecological and climatic conditions, *Hyalomma* species can complete their life cycle in one, two, or three hosts, with a duration of three months to over a year. Certain species like *H. schulzei* and *H. marginatum* are two-host ticks, while others, such as *H. truncatum* and *H. asiaticum*, are three-host ticks. *Hyalomma* ticks are widely distributed, and found in 21 Asian, six African, and 15 European countries [35]. The global spread of *Hyalomma* ticks is influenced by factors such as climate change, which enables these ticks to colonize new regions.

These ticks are vectors for a range of human pathogens, including *Rickettsia* spp. and the Crimean-Congo hemorrhagic fever (CCHF) virus. *Rickettsia aeschlimannii* and *Rickettsia sibirica mongolitimonae* have been isolated from various *Hyalomma* species. The CCHF virus, which was first identified in *Hyalomma* ticks in the 1960s, is transmitted by several species, particularly *H. marginatum* and *H. rufipes*, in regions across Asia, Africa, and Europe [36,37]. Based on all available reports, the highest risk of disease transmission occurs through *Hyalomma* spp. ticks, known to be the main vectors of Crimean Congo hemorrhagic fever, pose a significant biorisk in the region. In Türkiye, the main vector for CCHF is *H. marginatum*. Geographically, populations of *Hyalomma* spp. are widespread throughout rural areas in Türkiye. However, CCHF transmitted by *Hyalomma* spp. has become endemic in the rural areas of central Anatolia, and seasonal outbreaks of the disease are reported every year. The seasonal dynamics of *Hyalomma* spp. depend on the availability of suitable hosts and environmental conditions such as temperature and humidity [38,39].

According to the results, *Ixodes* spp. is the second most prevalent group of ticks that can infest humans in Türkiye. These ticks are known as three-host ticks, spending their immature stages on birds, hedgehogs, and rodents. After maturing, they can infest humans, cattle, canines, and a wide range of mammals. Ticks in the *Ixodes* group are significant vectors of diseases, particularly the *Ixodes ricinus* group known as a vector of Lyme borreliosis, tick-borne encephalitis, rickettsiosis, and human babesiosis.

To date, there are limited reports about clinical Lyme borreliosis in Türkiye. Only 50% of 84 reported Lyme patients were correctly diagnosed with Lyme disease from 1990 to 2022 [40]. Several studies have demonstrated that other *Ixodes* spp.-borne diseases, such as rickettsiosis, tick-borne encephalitis, and babesiosis, also occur in humans in Türkiye [41].

*Rhipicephalus* spp. ticks are typically two- or three-host ticks, except for the *Boophilus* group, which consists of one-host ticks. The *Rhipicephalus* genus contains more than 84 species, but human infestations have been reported with only three species from the *Rhipicephalus* group and two species from the *Boophilus* group in Türkiye. These ticks are potential vectors for several pathogens, including *Rickettsia* spp., *Anaplasma* spp., *Coxiella burnetii*, and *Ehrlichia* spp. [42]. There are a few reports available regarding tick-borne diseases in the region; however, most studies are limited to seroprevalence data and do not directly address tick bites [11].

*Haemaphysalis* spp. is one of the largest genera of ticks, comprising 167 species. Most known species are three-host ticks. According to our systematic review, six species of *Haemaphysalis* spp. infest humans in Türkiye. The vector competence of these species remains controversial, but reports indicate the presence of various rickettsial agents in *H. parva* and *H. sulcata.* [43,44].

*Dermacentor* species are three-host genera of ticks that have 35 species and are distributed worldwide. Mature *Dermacentor* spp. feed on medium and large mammals; immature forms prefer small mammals for infestation [43]. Based on the results, at least three species of *Dermacentor* spp. infested humans in Türkiye. They are medically important ticks and vectors of various pathogens. Specifically, *Dermacentor reticulatus* is the main vector of Omsk Hemorrhagic Fever and various *Rickettsial* agents. There are no reports of diseases caused by *Dermacentor* spp. bites in the region [45].

There are a few cases of human tick biting because of argasid ticks. Argasids are multi-host ticks and human infestation probability is low because they are mostly nocturnal, feed briefly on their host, and use an ambush strategy (nidicolous behavior) [43]. It is difficult to find argasid ticks on humans and there are more than 185 reported species. However, argasid ticks can play the role of vectors of pathogens. One of the most important pathogens transmitted with *Ornithodorus* is relapsing fever borreliosis in human populations yet these diseases have not been reported in Türkiye [46].

## Conclusion

More than 40 species of ticks could play a role in human tick-biting in Türkiye [4,21,22,29,31,41,44]. Some of these species, known as potential vectors of critical vector-borne diseases, can threaten community health [32,37,47]. Moreover, there are several reports about the role of these ticks in vector-borne diseases in Türkiye [21,23,47]. Therefore, it is predicted that new vector-borne infections such as babesiosis, novel rickettsial diseases, and tick-borne encephalitis may occur in the following years depending on the ecological conditions and the occurrence of new tick species, which can gain vectorial competence and pathogen adaptation. [11,48]. We recommend screening pathogens in ticks in surveillance studies. Additionally, the public should be informed about the local ticks and tick-borne diseases. In highly endemic or local risk areas, tick habitats can be flagged to warn individuals. Recent studies have reported the presence of the CCHF virus or a part of the virus genome in other tick species besides *H. marginatum*. These species include *Dermacentor marginatus, Rhipicephalus bursa, Rhipicephalus turanicus, Hyalomma excavatum, Haemaphysalis parva*, and *Ixodes ricinus* which have also been reported to bite humans [47,49]. This highlights the potential risk of these tick species in the transmission of CCHF and other tick-borne diseases in Türkiye [47]. In addition to the high prevalence of *Hyalomma* spp., the increasing number of cases involving *Ixodes* spp. also poses a risk for diseases caused by *Rickettsia* and *Spirochaetales*, such as Lyme disease. [50–53].

Ticks belonging to the *Hyalomma* and *Ixodes* genera are indeed known to be the most important vectors of tick-borne diseases in Türkiye [47,53]. *Hyalomma* ticks are particularly important as vectors of the CCHF virus. In contrast, *Ixodes* ticks are known to transmit Lyme disease and other tick-borne infections such as tick-borne encephalitis virus. For protection against these two vectors, educational posters and videos can be provided in endemic areas to inform the public about the prevention strategies and necessary measures in cases of *Hyalomma* and *Ixodes* biting. Additionally, mobile tools of disease risk assessment can be developed to identify tick species and related disease risks, especially in remote areas, where access to healthcare specialists is limited. Other tick genera such as *Dermacentor*, *Rhipicephalus*, *Haemaphysalis*, *Argas*, and *Ornithodorus* have also been reported as vectors or potential vectors of human diseases in Türkiye. However, these ticks’ prevalence and vectorial capacity may vary depending on the geographic region, climate, and host preferences.

Some of the human biting reports in Türkiye were not in line with tick fauna in Türkiye [54] due to differences in tick collection and identification methodologies. It is important to ensure the accuracy of tick species identification in human biting reports in Türkiye. Incorrect identification can lead to unreliable data and inaccurate statistics. Inconsistent reporting of tick species may also make comparing data across studies and regions difficult. 3.39% of ticks were not identified because standardized methods were not used. This number is higher than the number of the least common four species combined. Although the numbers of *Hyalomma* spp., *Ixodes* spp., and *Rhipicephalus* spp. might not be affected, the number of ticks in the other genera might dramatically change after the identification of unidentified ticks. Therefore, it is recommended to use standardized methods for tick identification and reporting to ensure the reliability of the data.

## Supporting information

S1 Appendix PRISMA 2020 Checklist. (DOCX)

S2 Appendix Numbers and percentages of tick bites on humans in Türkiye. (DOCX)

S3 Appendix Number of reported tick bites in selected studies. (XLSX)
