# Supplementary material for: Human tick biting and tick-borne disease risk in Türkiye: Systematic review
Source: PLoS Negl Trop Dis. 2025 Jun 9;19(6):e0013092. doi: 10.1371/journal.pntd.0013092 (PMC12176299; doi:10.1371/journal.pntd.0013092)
Supplement: S2 Appendix — (DOCX) [file pntd.0013092.s002.docx]

**S1 Table. Numbers and percentages of *Hyalomma spp.* tick bites on humans in Türkiye**

| Species | Number | Percentage |
| --- | --- | --- |
| *Hyalomma* spp. | 4196 | 17% |
| *Hyalomma* larvae | 308 | 1% |
| *Hyalomma* nymph | 11900 | 47% |
| *Hy. marginatum* | 4599 | 18% |
| *Hy. aegyptium* | 2482 | 10% |
| *Hy. detritium* | 332 | 1% |
| *Hy. anatolicum* | 366 | 1% |
| *Hy. doromedari* | 25 | 0% |
| *Hy. turanicum* | 229 | 1% |
| *Hy. isaaci* | 67 | 0.26% |
| *Hy. excavatum* | 738 | 3% |
| *Hy. impeltatum* | 2 | 0.01% |
| *Hy. rufipes* | 14 | 0.06% |
| *Hy. franchinii* | 33 | 0.13% |
| *Hy. scupense* | 30 | 0.12% |
| Total | **25321** | 100% |

**S2 Table. Numbers and percentages of *Ixodes spp.* tick bites on humans in Türkiye**

| Species | Number | Percentage |
| --- | --- | --- |
| *Ixodes* spp. | 303 | 2% |
| *Ixodes* larvae | 585 | 4% |
| *Ixodes* nymph | 7590 | 49% |
| *I. ricinus* | 6721 | 44% |
| *I. laguri* | 18 | 0.12% |
| *I. redikorzevi* | 20 | 0.13% |
| *I. hexagonus* | 6 | 0.04% |
| *I. frontalis* | 41 | 0.27% |
| *I. acumınatus* | 43 | 0.28% |
| *I. gibbosus* | 25 | 0.16% |
| Total | **15352** | 100% |

**S3 Table. Numbers and percentages of *Haemaphysalis spp.* tick bites on humans in Türkiye**

| Species | Number | Percentage |
| --- | --- | --- |
| *Haemaphysalis* spp. | 698 | 24% |
| *Haemaphysalis* larvae | 1 | 0.03% |
| *Haemaphysalis* nymph | 66 | 2% |
| H. sulcata | 62 | 2% |
| H. concinna | 22 | 1% |
| H. inermis | 4 | 0.14% |
| *H. erinacei* | 4 | 0.14% |
| *H. parva* | 1813 | 63% |
| *H. punctata* | 207 | 7% |
| Total | **2877** | 100% |

**S4 Table. Numbers and percentages of *Rhipicephalus spp.* tick bites on humans in Türkiye**

| Species | Number | Percentage |
| --- | --- | --- |
| *Rhipicephalus spp.* | 1026 | 15% |
| *Rhipicephalus larvae* | 31 | 0.44% |
| *Rhipicephalus nymph* | 414 | 6% |
| *R. sanguineus* | 1438 | 20% |
| *R. bursa* | 1925 | 27% |
| *R. turanicus* | 2156 | 31% |
| *R. (B) kohlsi* | 2 | 0.03% |
| *R. (B) annulatus* | 47 | 1% |
| Total | **7039** | 100% |

**S5 Table. Numbers and percentages of *Dermacentor spp.* tick bites on humans in Türkiye**

| Species | Number | Percentage |
| --- | --- | --- |
| *Dermacentor* spp. | 199 | 14% |
| *Dermacentor* nymph | 102 | 7% |
| *D. marginatus* | 994 | 71% |
| *D. daghestanicus* | 14 | 1% |
| *D. niveus* | 79 | 6% |
| *D. reticulatus* | 14 | 1% |
| Total | **1402** | 100% |

**S6 Table. Numbers and percentages of soft tick bites on humans in Türkiye**

| Species | Number | Percentage |
| --- | --- | --- |
| *Ornithodoros* spp. | 2 | 3% |
| *O. lahorensis* | 2 | 3% |
| *Argas* spp. | 28 | 47% |
| *Argas persicus* | 23 | 38% |
| *Argas reflexus* | 3 | 5% |
| *Otobius megnini* | 2 | 3% |
| Total | **60** | 100% |
